# Prevalence, potential determinants, and treatment-seeking behavior of acute respiratory infection among children under age five in India: Findings from the National Family Health Survey, 2019-21

**DOI:** 10.1186/s12890-023-02487-4

**Published:** 2023-06-06

**Authors:** Jesty Saira Varghese, T. Muhammad

**Affiliations:** 1 Indian Institute of Technology (IIT) Delhi, New Delhi, 110016 India; 2 Department of Family & Generations, International Institute for Population Sciences (IIPS), Mumbai, 400088 Maharashtra India

**Keywords:** ARI, Respiratory tract diseases, Treatment-seeking, Children, India

## Abstract

**Background:**

Acute respiratory infections (ARI) are a major cause of mortality and morbidity among under-five children worldwide, particularly in developing countries. Current evidence using nationally representative data on determinants and care-seeking behavior for ARI is limited in the Indian context. Hence, the present study complements the existing literature by examining the prevalence, determinants, and health-care-seeking behavior regarding ARI among Indian children under age five.

**Study design:**

Cross-sectional study.

**Methods:**

The data for the present study were drawn from the fifth round of the National Family Health Survey (NFHS-5) conducted in 28 states and 8 union territories of India in 2019-21. A total of 222,233 children age less than five years were selected to estimate the prevalence and determinants of ARI, and 6198 children having ARI were selected to explore the treatment-seeking behavior. Bivariate analysis and multivariable binary logistic regression analysis were employed.

**Results:**

Among children under five years, 2.8% suffered from ARI in the two weeks preceding the survey, and 56.1% sought treatment for ARI. Younger age, a recent episode of diarrhea, maternal asthmatic history, and tobacco smoke exposure in the household increase the risk of having ARI. Further, having a separate room as a kitchen in the household reduces the likelihood of having ARI by 14% (AOR: 0.86; CI: 0.79–0.93). Female children (AOR: 0.88; CI: 0.77-1.00) and children belonging to households having difficulty in accessing transport to health facility (AOR: 0.83; CI: 0.69–0.99) are less likely to seek treatment.

**Conclusion:**

The study identified several socio-demographic, maternal, and household characteristics associated with ARI and treatment seeking for ARI. The study also recommends making health centers more accessible to the people in terms of proximity and cost.

## Background

Acute respiratory infection (ARI) is an infection of the respiratory tract that interferes the normal breathing of an individual [1]. ARI is classified into Upper Respiratory Infection (URI) and Lower Respiratory Infection (LRI) [2]. They are a major cause of mortality and morbidity among under-five children worldwide, particularly in developing countries [3]. For instance, according to the Global Burden of Disease Study 2019, lower respiratory infections are the second leading cause of death among children under five years old [4]. Highlighting the severity of the disease, the World Health Organization (WHO) and United Nations (UN) suggested that ARIs should be addressed as ‘presumed pneumonia’ [5]. In an effort to reduce deaths due to pneumonia, the Global Action Plan for Prevention and Control of Pneumonia was developed in 2009 [6]. However, since pneumonia and diarrhea share the same determinants, it was recognized that the prevention and control strategies of both diseases should be coordinated. Accordingly, in 2013, the Integrated Global Action Plan for Pneumonia and Diarrhea was implemented with an aim to end preventable child deaths due to pneumonia and diarrhea by 2025 [7]. Despite the global strategies to end pneumonia deaths, over two-thirds of the worldwide burden of pneumonia and diarrhea mortality occur in just fifteen countries. Further, nearly half a million pneumonia and diarrhea deaths occur in just two countries- India and Nigeria. In India, the death rate in children under five years (per 1000 live births) due to pneumonia was 6.3 in 2016 [8].

Identifying the risk factors of ARI is imperative to reduce the mortality and morbidity burden due to ARI. Accordingly, previous studies have identified several household, maternal, and child characteristics as risk factors for the infection. These include age, gender, nutritional status, and household income [9–13, 15]. Earlier evidence suggests that the incidence of ARI is higher among older, male children [9–11] Further, malnourished children and those from poor households have a higher risk of contracting the infection [12, 13, 15]. Studies have also found that children who suffered from diarrhea recently have a higher likelihood of developing ARI [3, 16]. The adverse impact of the use of solid fuels for cooking on the incidence of respiratory tract infections has been established in a multitude of studies conducted in India [14, 15, 17, 18] The detrimental effect of the alternate sources of indoor air pollution, including the unavailability of a separate room as kitchen and second-hand smoke exposure, has been highlighted in a cross-sectional study in India [18]. Further, a quantitative systematic review of studies from developed countries estimated that handwashing reduces the incidence of respiratory infections [19]. Earlier evidence also suggests that ambient humidity has a significant impact on respiratory diseases, thus leading to increased incidence of the disease in winter season [20, 21]. Further, a cross-sectional study conducted among young children in Brazil revealed that household crowding places children more susceptible to acute lower respiratory tract infection [22].

Caretakers play a pivotal role in recognizing the symptoms of the infection and providing appropriate care immediately [23, 24] Timely provision of a full course of antibiotics is imperative in preventing pneumonia deaths [24]. ‘Health seeking ‘behaviour’ is governed by a multitude of factors, including socioeconomic, sociocultural, and demographic factors [25, 26]. Previous studies have documented the positive influence of maternal education on health care seeking behaviour [25, 27]. Earlier evidence also suggests that socioeconomic status, family size, and distance to health facility influences health care seeking behaviour [10, 23, 25, 27, 28].

The prevalence of ARI in India remains almost unchanged between the fourth (2014-15) and fifth (2019-21)- rounds of NFHS (NFHS-4: 2.73%, NFHS-5: 2.79%), thus remaining a public health challenge [29, 30]. Limited studies have focused on the risk factors of the infection using nationally representative data. [3, 18, 31, 32]. However, these studies have ignored the asthmatic history of mothers as a possible predictor of ARI. A case-control study in Calcutta found that children have a two to three-fold higher risk of having ARI if their parents or siblings have an asthmatic history. [14] Asthmatic history among family members can aid in the prevention and early diagnosis of ARI. Hence, in this study, in addition to the other factors, we investigate whether ARI among children is associated with maternal asthmatic history. We have also considered additional factors, including seasonal variation and handwashing habits, neglected in previous population-based studies in India. Further, to attain the Sustainable Development Goal (SDG) of ending preventable deaths of children aged under five years, it is imperative to reduce the morbidity due to ARI and ensure timely treatment [33]. Current evidence using nationally representative data on household care-seeking behaviour for ARI is limited in the Indian context. Hence, the present study complements and augments the existing literature by examining the prevalence, determinants, and health-care seeking behaviour regarding ARI, utilizing the most recent round of the National Family Health Survey (NFHS-5) conducted in 2019-21.

## Methods

### Data

**Fig. 1:**
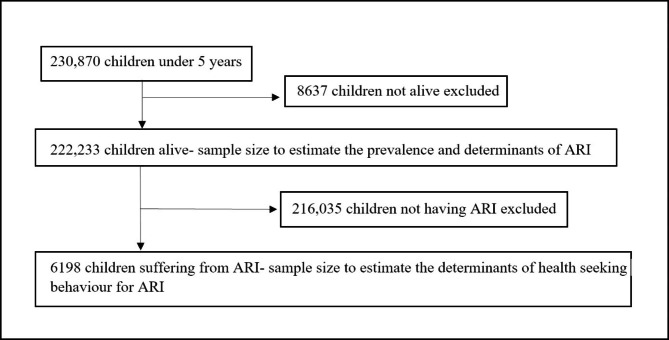
Selection of study participants

The data for the present study was drawn from the fifth round of the NFHS conducted in all 28 states and 8 union territories of India in 2019-21. NFHS is a large-scale, multi-round survey conducted in a representative sample of households throughout India. NFHS surveys have been conducted under the stewardship of the Ministry of Health and Family Welfare, Government of India, and the International Institute of Population Sciences (IIPS), Mumbai was the nodal agency. The survey provides crucial information on women and child health, their socio-economic characteristics, nutrition, lifestyle, and other vital factors. NFHS-5 adopted a two-stage stratified sampling design. Detailed information on the sampling strategy is available in the report [29]. The sample size for estimating the prevalence and determinants of ARI includes 222,233 children aged less than five years. 6198 children who had symptoms of ARI in the two weeks preceding the survey were considered for the objective of examining the determinants of health care seeking behaviour for ARI (Fig. 1). The analysis is based on secondary data available in the public domain for research; thus, no ethical approval was required. Further, there is no identifiable information of individuals in the data.

### Variable description

#### Dependent variable

There are two dependent variables in this study- whether the child had ARI and whether the child received treatment for ARI. During the UNICEF/WHO meeting on child-survival survey-based indicators, held in New York, June 17–18, 2004, the definition of ARI to be used in the Multiple Indicator Cluster Surveys (MICS) was chosen and is based on ‘mother’s perceptions of a child who has a cough, is breathing faster than usual with short, quick breaths or is having difficulty breathing, excluding children that had only a blocked nose [34]. In NFHS-5, mothers were asked if their children under five years had been ill with a cough in the two weeks preceding the survey accompanied by short and rapid breathing that was chest related. If the answer was yes, the outcome variable was coded as 1, meaning “Having ARI”; else, it was coded as 0, "Not having ARI". Regarding treatment seeking behaviour, the outcome variable was coded as 1 if treatment was sought from a health facility/provider and 0 otherwise. Health facility/provider excludes traditional healer, friend or relative [29].

#### Explanatory variables

The explanatory variables were derived from the literature, and they include the following:

Determinants of ARI.

***Child-related factors-*** The characteristics of children included age (0–11,12–23,24–35,36–47, and 48–59 months), sex (male, female), religion (Hindu, Muslim, Others), caste (scheduled caste, scheduled tribe, or other castes), nutritional status (stunted, not stunted), birth order (less than or equal to three, greater than three), and incidence of diarrhea (no,yes) [3, 10, 35]. Children whose height-for-age Z score was below minus two standard deviations from the median of the reference population were considered stunted.

***Household-related factors-*** The household factors considered are the place of residence (urban, rural), wealth index (from poorest to richest), fuel used for cooking (solid fuel, clean fuel), frequency household members smoke in the house (never, daily/weekly, and monthly/less than once a month), availability of a separate room as kitchen, household crowding and handwashing habits [10, 18, 35]. Kerosene, coal/lignite, charcoal, wood, straw/shrubs/grass, agricultural crop waste, and dung cakes were classified as solid fuels, whereas electricity, liquified petroleum gas/natural gas, and biogas were classified as clean fuels [18]. Household crowding was measured based on the number of persons sleeping per room. If more than three persons slept per room, the household was categorised as crowded. Handwashing habits were measured based on whether a separate place of handwashing was observed in the household or not.

***Maternal factors***- The characteristics of mothers included the education level (literate, illiterate), history of respiratory illness including asthma, and mass media exposure [10, 36]. In NFHS-5, women were asked about how often they read a newspaper or magazine, listened to the radio, watched television, or went to the cinema. Those who responded at least once a month for cinema and once a week for other mass media were considered as regularly exposed to that form of media. Respondents who have regular exposure to at least one form of media were classified as ‘exposed’ and others as ‘not exposed’ [29].

***Other factors***- In addition to these factors, the season of the interview was considered and was classified as winter (October-January), summer (February-May), and rainy (June-September) [37].

Determinants of treatment-seeking for ARI.

Major explanatory variables included child’s age (0–11,12–23,24–35,36–47, and 48–59 months), sex (male, female), religion (Hindu, Muslim, Others), caste (scheduled caste, scheduled tribe, or other castes), birth order ( < = 3, > 3), place of residence (rural, urban), wealth index (poorest/poorer, middle, richer/richest), household size (< 6, >=6), distance to health facility (no/not a big problem, big problem), having to take transport (no/not a big problem, big problem), mother’s education (no education, primary, secondary, higher) and exposure to mass media (exposed, not exposed) [10, 23].

### Statistical analysis

Descriptive statistics and bivariate analysis were employed to present the prevalence of ARI.. Since the dependent variables are dichotomous, multivariable binary logistic regression analysis was performed to determine the determinants of ARI and treatment seeking for the infection. The results are presented as adjusted odds ratios (AOR) with a 95% confidence interval (CI). All the statistical analyses were performed using Stata 16 software [38]. Appropriate weights were used to ensure the representativeness of the sample.

## Results

Out of the children aged less than five years, 2.8% suffered from ARI in the two weeks preceding the survey (Table 1). Delhi (5.6%), Ladakh (5.3%), Puducherry (4.9%), Meghalaya (4.8%), and Jammu and Kashmir (3.9%) have the highest ARI prevalence. The lowest rates were observed in Dadra & Nagar Haveli and Daman & Diu (0.3%) followed by Chandigarh (0.3%), Mizoram (0.6%), Sikkim (0.7%) and Goa (0.9%).

**Table 1:** Prevalence of ARI in the states/UTs of India, 2019-21

Serial Number	State	ARI (%) (95% CI)	Serial Number	State	ARI (%) (95% CI)
1	Andaman and Nicobar	1.7 (0.0-5.7)	19	Lakshadweep	1.4 (0.0-9.1)
2	Andhra Pradesh	2.4 (2.0–2.8)	20	Madhya Pradesh	2.6 (2.3–2.9)
3	Arunachal Pradesh	2.1 (0.0-4.3)	21	Maharashtra	3.2(2.9–3.4)
4	Assam	2.5 (2.1–2.9)	22	Manipur	1.8 (0.5–3.0)
5	Bihar	3.5 (3.3–3.8)	23	Meghalaya	4.8 (3.4–6.3)
6	Chandigarh	0.3 (0.0-1.3)	24	Mizoram	0.6 (0.0-1.7)
7	Chhattisgarh	1.5 (1.1–1.8)	25	Nagaland	1.1 (0.0-2.5)
8	Dadra & Nagar Haveli and Daman & Diu	0.3 (0.0-1.5)	26	Odisha	3.2 (2.7–3.6)
9	Delhi	5.6 (4.8–6.4)	27	Puducherry	4.9 (1.4–8.4)
10	Goa	0.9 (0.0-2.3)	28	Punjab	2.5 (2.0–2.9)
11	Gujarat	1.0 (0.8–1.2)	29	Rajasthan	2.9 (2.7–3.2)
12	Haryana	2.3 (1.8–2.7)	30	Sikkim	0.7 (0.0-2.9)
13	Himachal Pradesh	1.5 (0.7–2.2)	31	Tamil Nadu	1.1 (0.9–1.3)
14	Jammu and Kashmir	3.9 (2.9–4.8)	32	Tripura	1.3 (0.4–2.2)
15	Jharkhand	2.1 (1.8–2.4)	33	Uttar Pradesh	3.5 (3.4–3.7)
16	Karnataka	1.5 (1.3–1.8)	34	Uttarakhand	2.3 (1.6–3.0)
17	Kerala	2.4 (2.0–2.9)	35	West Bengal	2.8 (2.6–3.1)
18	Ladakh	5.3 (0.0-14.0)	36	Telangana	2.2 (1.8–2.6)
				**INDIA**	**2.8(2.7–2.9)**

*CI-* Confidence Interval

Table 2 presents the distribution of ARI among under-five children in India according to their background characteristics and the multivariable binary logistic regression results to determine the predictors of ARI. ARI was more prevalent in children aged 0–11 months (3.4%) compared to those aged 12–23 months (3.3%), 24–35 months (2.7%), 36–47 months (2.5%) and 48–59 months (2.1%). Male children (3.0%) were more susceptible to ARI than female children (2.5%). ARI prevalence was high among children belonging to the scheduled caste (3.0%) when compared to those children belonging to the scheduled tribe (2.4%) and other castes (2.8%). Children from rural areas and poor households had a higher prevalence of ARI than their counterparts.

**Table 2:** Determinants of ARI among children under age five in India

Variable	Sample characteristics (children in the study)	Children suffering from ARI in the last two weeks	Multivariable binary logistic regression
	Number (Percent)	Number (Percent)	AOR (95% CI) (p value)
**Child’s age (in months)**			
0–11	44,493(20.0)	1489 (3.4)	1.43 (1.28–1.60) (< 0.001)
12–23	43,247(19.5)	1429 (3.3)	1.41 (1.26–1.58) (< 0.001)
24–35	43,916(19.8)	1193 (2.7)	1.23 (1.09–1.38) (< 0.001)
36–47	44,075(19.8)	1104 (2.5)	1.16 (1.03–1.30) (0.013)
48–59	46,503(20.9)	983 (2.1)	1.00
**Child’s sex**			
Male	115,196(51.8)	3480 (3.0)	1.00
Female	107,037(48.2)	2718 (2.5)	0.84 (0.79–0.90) (< 0.001)
**Child’s nutritional status**			
Stunted	71,566(32.2)	2101 (2.9)	1.00
Not stunted	130,185(58.6)	3651 (2.8)	0.99 (0.92–1.07) (0.884)
Missing	20,481(9.2)	445 (2.2)	0.79 (0.69–0.91) (0.001)
**Whether child had diarrhea recently**			
No	205,645(92.5)	4807 (2.3)	1.00
Yes	16,213(7.3)	1386 (8.6)	3.67 (3.38–3.99) (< 0.001)
Don’t know	375(0.2)	5 (1.3)	0.61 (0.24–1.52) (0.288)
**Child’s birth order**			
Less than/equal to 3	196,129(88.3)	5369 (2.7)	1.00
Greater than 3	26,103(11.8)	829 (3.2)	1.08 (0.98–1.19) (0.139)
**Mother’s education**			
Not educated	46,635(21.0)	1343 (2.9)	1.00
Educated	175,597(79.0)	4855 (2.8)	1.09 (0.99–1.19) (0.075)
**Mother had respiratory diseases/asthma**			
Yes	1958(0.9)	138 (7.0)	1.00
No	220,275(99.1)	6060 (2.8)	2.48 (1.91–3.21) (< 0.001)
**Mother’s exposure to mass media**			
Yes	116,338(52.4)	3080 (2.7)	1.00
No	105,895(47.7)	3117 (2.9)	1.03 (0.95–1.11) (0.481)
**Type of fuel used for cooking**			
Solid fuel	103,785(46.7)	3112 (3.0)	1.00
Clean fuel	105,884(47.7)	2751 (2.6)	0.94 (0.86–1.02) (0.126)
Not resident/no food cooked in home	12,563(5.7)	335 (2.7)	0.86 (0.74–1.01) (0.062)
**Smoke exposure in the household**			
Never	120,699(54.3)	2989 (2.5)	1.00
Daily/weekly	81,065(36.5)	2535 (3.1)	1.22 (1.14–1.31) (< 0.001)
Monthly/less than once a month	20,466(9.2)	673 (3.3)	1.28 (1.14–1.43) (< 0.001)
**Separate kitchen in the household**			
No	60,635(27.3)	1991 (3.3)	1.00
Yes	161,598(72.7)	4206 (2.6)	0.86 (0.79–0.93) (< 0.001)
**Household crowding**			
Not crowded ( < = 3 persons sleep in a room)	130,139(58.6)	3561 (2.7)	1.00
Crowded (> 3 persons sleep in a room)	92,094(41.4)	2637 (2.9)	0.98 (0.91–1.05) (0.557)
**Place of handwashing in household**			
Not observed	8184(3.7)	257 (3.1)	1.00
Observed	214,049(96.3)	5941 (2.8)	0.92 (0.77–1.09) (0.339)
**Season of interview**			
Winter	98,122(44.2)	2952 (3.0)	1.00
Summer	34,757(15.6)	853 (2.5)	0.83 (0.75–0.91) (< 0.001)
Rainy	89,353(40.2)	2393 (2.7)	0.82 (0.75–0.88) (< 0.001)
**Wealth Quintile**			
Poorest	53,817(24.2)	1723 (3.2)	1.00
Poorer	47,984(21.6)	1472 (3.1)	0.95 (0.86–1.04) (0.265)
Middle	43,519(19.6)	1167 (2.7)	0.85 (0.75–0.95) (0.005)
Richer	41,242(18.6)	993 (2.4)	0.79 (0.69–0.91) (0.001)
Richest	35,671(16.1)	844 (2.4)	0.85 (0.72–0.99) (0.038)
**Religion**			
Hindu	176,335(79.4)	4927 (2.8)	1.00
Muslim	36,163(16.3)	1000 (2.8)	1.01 (0.91–1.12) (0.881)
Others	9735(4.4)	270 (2.8)	1.12 (0.96–1.29) (0.152)
**Caste**			
Scheduled caste	51,395(23.1)	1551 (3.0)	1.00
Scheduled tribe	22,135(10.0)	532 (2.4)	0.76 (0.67–0.85) (< 0.001)
Others	136,623(61.5)	3770 (2.8)	0.98 (0.91–1.07) (0.708)
Missing/don’t know	12,080(5.4)	345 (2.9)	1.03 (0.86–1.23) (0.772)
**Place of Residence**			
Urban	59,780(26.9)	1378 (2.3)	1.00
Rural	162,452(73.1)	4820 (3.0)	1.19 (1.08–1.32) (0.001)
**TOTAL**	**222,233**	**6198 (2.8)**	
Pseudo R [2]			0.0351

*CI-* Confidence Interval, *AOR-* Adjusted Odds Ratio

The multivariable binary logistic regression analysis results (Table 2) reveal that children who recently suffered from diarrhea have 3.67 times (AOR:3.67; CI: 3.38 to 3.99) higher odds of suffering from ARI. In comparison to children aged 48–59 months, children aged 0–11, 12–23. 24–35 and 36–47 months have 43% (AOR:1.43; CI: 1.28 to 1.60), 41% (AOR:1.41; CI: 1.26 to 1.58), 23% (AOR:1.23; CI: 1.09 to 1.38) and 16% (AOR:1.16; CI: 1.03 to 1.30) higher odds of having ARI, respectively. Female children were 16% (AOR:0.84; CI: 0.79 to 0.90) less likely to suffer from ARI. Children belonging to rural areas have 19% (AOR:1.19; CI:1.08 to 1.32) higher odds of suffering from ARI. Considering the wealth status, the odds of the child suffering from ARI reduces with increasing wealth status of the household.

Considering the maternal characteristics, asthmatic history of mothers was found to be significant risk factor of ARI. If mothers have any respiratory disease, including asthma, their children have 2.48 times (AOR:2.48; CI:1.91 to 3.21) higher odds of having the disease. Compared to the winter season, the likelihood of developing ARI is lower among children, if the interview was conducted in the summer (AOR:0.83; CI: 0.75 to 0.91) or during rainy (AOR:0.82; CI: 0.75 to 0.88) season. Smoke exposure in the household was found to be a significant risk factor of ARI. Compared to no smoke exposure, the likelihood of having the infection increased with an increase in the frequency of smoke exposure inside the household (Daily/weekly exposure- AOR:1.22; CI: 1.14 to 1.31; Monthly/less than once a month- AOR:1.28; CI: 1.14–1.43). Further, having a separate room as a kitchen in the household reduces the likelihood of having ARI by 14% (AOR:0.86; CI: 0.79 to 0.93).

Table 3 presents the percentage of children receiving treatment for ARI and the factors influencing the treatment seeking behaviour. About 56% of the children suffering from ARI sought treatment from a health facility/ provider. The logistic regression analysis revealed that gender of the child, religion, caste and problems in taking transport are significantly associated with the treatment seeking for ARI. Female children are 12% (AOR: 0.88; CI: 0.77 to 1.00) less likely to receive treatment compared to male children. Further, Muslim children had 26% (AOR: 1.26; CI: 1.03 to 1.55) higher chances of receiving treatment compared to children belonging to Hindu religion. Moreover, children belonging to households where having to take transport was a big problem are 17% (AOR: 0.83; CI: 0.69 to 0.99) less likely to seek treatment.

**Table 3:** Factors associated with treatment seeking for ARI among children under age five in India

Variable	Sample characteristics (children suffering from ARI)	Children suffering from ARI receiving any treatment	Multivariable binary logistic regression
	Number (Percent)	Number (Percent)	AOR (95% CI) (p value)
**Age of child (in months)**			
0–11	1489 (24.0)	833 (55.9)	1.00
12–23	1429 (23.1)	853 (59.7)	1.17 (0.96–1.41) (0.115)
24–35	1193 (19.3)	655 (54.9)	0.94 (0.78–1.15) (0.562)
36–47	1104 (17.8)	591 (53.5)	0.91 (0.74–1.11) (0.356)
48–59	983 (15.9)	548 (55.8)	0.99 (0.80–1.23) (0.957)
**Child’s sex**			
Male	3480 (56.2)	2003 (57.6)	1.00
Female	2718 (43.9)	1476 (54.3)	0.88 (0.77-1.00) (0.052)
**Child’s birth order**			
Less than/equal to 3	5369 (86.6)	3013 (56.1)	1.00
Greater than 3	829 (13.4)	466 (56.2)	1.01 (0.82–1.23) (0.956)
**Religion**			
Hindu	4927 (79.5)	2733 (55.5)	1.00
Muslim	1000 (16.1)	610 (61.0)	1.26 (1.03–1.55) (0.023)
Others	270 (4.4)	136 (50.2)	0.86 (0.64–1.15) (0.301)
**Caste**			
Scheduled caste	1551 (25.0)	885 (57.0)	1.00
Scheduled tribe	532 (8.6)	272 (51.1)	0.77 (0.61–0.98) (0.035)
Others	3770 (60.8)	2124 (56.4)	0.95 (0.81–1.12) (0.562)
Missing/don’t know	345 (5.6)	198 (57.5)	0.89 (0.62–1.27) (0.493)
**Place of residence**			
Urban	1378 (22.2)	766 (55.6)	1.00
Rural	4820 (77.8)	2713 (56.3)	1.02 (0.84–1.23) (0.868)
**Wealth index**			
Poorest/poorer	3194 (51.5)	1842 (57.7)	1.00
Middle	1167 (18.8)	642 (55.0)	0.87 (0.72–1.05) (0.150)
Richer/richest	1836 (29.6)	995 (54.2)	0.84 (0.69–1.02) (0.078)
**Household size**			
Less than 6	2859 (46.1)	1658 (58.0)	1.00
Greater than/equal to 6	3339 (53.9)	1821 (54.6)	0.87 (0.76–0.99) (0.047)
**Distance to health facility**			
No/not a big problem	4293 (69.3)	2447 (57.0)	1.00
Big problem	1905 (30.7)	1031 (54.1)	0.98 (0.82–1.17) (0.814)
**Having to take transport**			
No/not a big problem	4417 (71.3)	2535 (57.4)	1.00
Big problem	1781 (28.7)	944 (53.0)	0.83 (0.69–0.99) (0.048)
**Maternal education**			
No education	1343 (21.7)	761 (56.7)	1.00
Primary	915 (14.8)	517 (56.6)	1.02 (0.82–1.27) (0.866)
Secondary	3169 (51.1)	1783 (56.3)	1.06 (0.89–1.27) (0.520)
Higher	772 (12.5)	417 (54.0)	1.04 (0.80–1.35) (0.772)
**Mother’s exposure to mass media**			
No	3117 (50.3)	1781 (57.1)	1.00
Yes	3080 (49.7)	1698 (55.1)	1.86 (1.15–3.01) (0.586)
**Total**	**6198**	**3479 (56.1)**	
Pseudo R [2]			0.0087

*CI-* Confidence Interval, *AOR-* Adjusted Odds Ratio

## Discussion

**Fig. 2:**
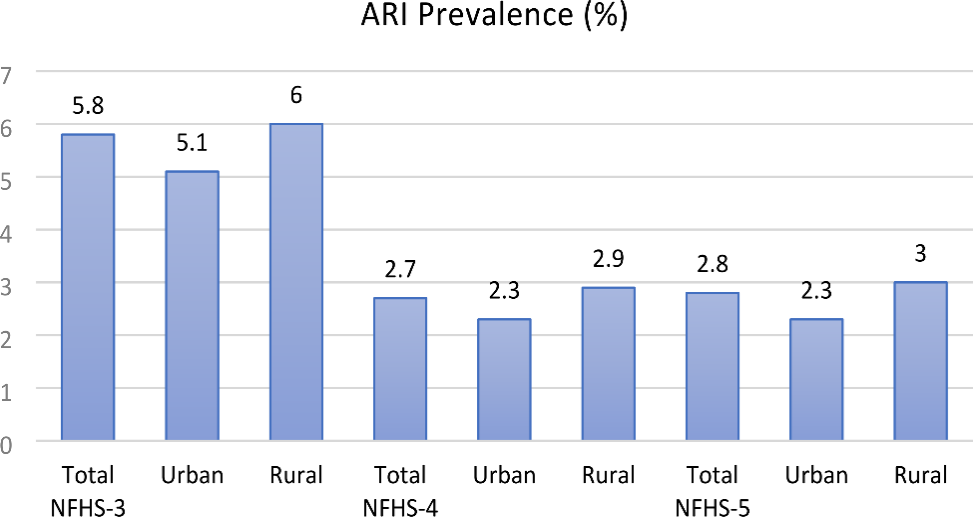
Prevalence of ARI in India from NFHS-3 (2005-06) to NFHS-5 (2019-21)

The present study was conducted to determine the prevalence, determinants, and treatment-seeking for ARI among under-five children in India. In this study, the prevalence of ARI was estimated to be 2.8%, which is similar to the previous round of the NFHS (NFHS 4-2.7%) [30]. However, this is a sharp decline from the figures estimated in 2005-06 (5.8%), during the third round of the survey [39] (Fig. 2**)**. The prevalence of ARI in India is similar to the overall prevalence in Bangladesh (3%), as reported by the Bangladesh Demographic and Health Survey (BDHS), 2017 − 18 [40]. A decline in ARI prevalence from NFHS-4 to NFHS-5 been observed in a few Indian states with the greatest percentage decline in Chandigarh (2.5%), Uttarakhand (2.4%), Tamil Nadu (1.7%), Punjab (1.7%), and Jammu and Kashmir (1.6%) [29, 30]. Further, the study found that various household, maternal and child characteristics are significantly associated with the prevelance of ARI.

Children who had diarrhea recently were more likely to suffer from the infection. A similar interrelationship was observed in recent studies conducted in India and Ethiopia [3, 16]. A recent episode of diarrhea could imply weakened immunity, and hence the child would be prone to further infections [16]. Moreover, the risk of suffering from ARI declines with increasing age of the children. Children aged between 48 and 59 months had lesser risk of contracting the infection compared to the younger age groups. These findings are consistent with a wide range of previous studies [10, 41−44]. This may be due to immature immune system among young age group children [34]. In line with the other studies, [3, 10, 36, 45−48] male children were found to be at a higher risk of the infection. A probable reason would be that the higher tendency of male children to play outside home gets them exposed to infected aerosols from the surrounding outdoor environment compared to female children [49]. Another reason for the high vulnerability of boys could be genetic or there could be higher reporting by the mothers for boys due to gender bias, which potentially causes mothers to notice symptoms of the disease among boys [10].

Amongst household factors, children belonging to rural areas and poorer households had a higher risk of ARI. Although air pollution is higher in urban areas which can increase the incidence of respiratory diseases, possible reason for our finding might be the poor socio-economic condition of the households in rural areas. Studies conducted in other low and middle-income countries have reported similar association [10, 21]. A multi-level analysis of the risk factors of ARI in Indonesia revealed that children of rich families were less vulnerable to ARI symptoms [35]. Similar findings were reported in other studies [5, 36] [44], [50]. Congruent with these findings, our study also estimated that children from poor socio-economic backgrounds are more likely to suffer from the infection. Further, our analysis indicated that the likelihood of having the infection increased with an increase in the frequency of smoke exposure inside the household and the unavailability of a separate room as a kitchen in the household. These findings are consistent with a previous study conducted in India based on the NFHS-4 dataset which concluded that the smoking behaviour of household members and the unavailability of a separate room as a kitchen in the household increases the risk of having ARI [18]. Household smoke exposure can expose children to passive smoke, thereby increasing the risk of respiratory infection [49]. Moreover, having a separate room as a kitchen can prevent children from inhaling the smoke, which becomes increasingly harmful if solid fuels are used for cooking [18]. However, the use of solid fuels for cooking and handwashing habits were not found to be associated with ARI. Although significant differences were observed in the bivariate analysis, the variables were not statistically significant in the logistic regression model, when adjusted for other covariates. A similar finding was observed by Harerimana et al. in a study done in Rwanda and Lutpiatina et al. in a study conducted in Indonesia [21, 35].

Considering maternal characteristics, maternal asthma was found to be associated with ARI. The association of maternal asthma as a significant risk factor of ARI among children, is consistent with the findings of other studies [12, 14, 36]. As children spent most of the time indoors, it makes them susceptible to contract the infection from any of the family members suffering from respiratory infection [49]. In line with the other studies [51, 52], this study also found that the likelihood of having ARI was higher if the interview was conducted in the winter season. A similar interrelationship was observed in a longitudinal, cohort study conducted in Gulbarga city in Karnataka [36]. Inhaling of cold air during the winter season can increase the susceptibility to respiratory tract infections [51]. Moreover, the low environmental temperatures might force people to stay indoors, leading to increased opportunities for the virus to spread among family members [36].

According to the present study, about 56% of the children suffering from ARI sought treatment from a health facility/provider. However, this is a sharp decline from the NFHS-4 estimates that treatment was sought for 78% of the children with ARI symptoms [30]. Phase 2 of the NFHS-5 survey, involving 14 states/UTs were conducted during 2nd January 2020–30th April 2021 [29]. The country was in a total/partial lockdown during the period. Studies have reported a decline in the proportion of people seeking medical treatment for acute health problems during the lockdown period [53, 54]. Hence, a possible reason for the decline in treatment-seeking behaviour for ARI would be due to the lockdown restrictions and the fear of contracting COVID-19 infection.

The study found that female children are less likely to receive treatment for ARI. This finding is consistent with a study conducted in India which reported that sex differentials exist in the health care seeking behaviour of parent and female children are less likely to receive treatment [25]. However, the finding is incongruent with other studies that reported no sex differentials or revealed that female children have a higher probability of receiving treatment [10, 55]. Consistent with the findings of Prakash (2014) [25], Muslim children were more likely to receive treatment compared to those belonging to Hindu religion. Problems in taking transport emerged as a factor negatively affecting the treatment seeking for ARI. Previous research has reported that long distance to health facility, lack of transportation and poor roads adversely influence the utilization of health services [23, 28, 56, 57].

## Strengths and limitations of the study

The present study used nationally representative, population-based survey data. Utilizing the most recent round of NFHS (2019-21) aids in gaining insights into the current levels of ARI in India and its states. This further helps in targeting interventions to reduce ARI. The study has also considered the association of ARI with factors such as maternal asthmatic history, seasonal variation, and handwashing habits, which were neglected in previous population-based studies conducted in India [10, 18]. Moreover, studies examining the determinants of health-seeking for ARI remain scare in the Indian context. Despite this, the study has certain limitations. Firstly, in this study, children were classified as having acute respiratory infection based on the signs and symptoms of ARI reported by mothers. The presence of ARI is not validated by medical personnel, which is a limitation of this study. Secondly, mother is asked about the signs and symptoms that the child experienced in the past two weeks. Hence the potential effect of recall bias on our results cannot be ignored. Thirdly, due to unavailability of information, the study did not explore the effect of ambient air pollution on the risk of ARI. Fourthly, factors at the community, district, and state level can influence our findings. Thus, we recommend future studies to adopt nested regression models or multilevel regression models. Finally, due to cross-sectional nature of the study, a cause-effect relationship could not be established.

## Conclusion

Despite several efforts by global agencies to reduce the incidence of ARI, they remain a paramount public health concern responsible for a wide range of mortality and morbidity among under-five children worldwide. This study identified several factors associated with ARI and its treatment-seeking behaviour among under-five children in India. Potential areas for intervention highlighted by this study include community campaigns highlighting the symptoms and treatment of ARI, to ensure that the infection is diagnosed early and deaths are averted.

The study found that the odds of having ARI increase with an increase in the frequency of smoke exposure and the unavailability of a separate kitchen in the household. Therefore, in addition to existing anti-tobacco campaigns and tobacco quitline services, authorities should promote campaigns to make people aware of the adverse impact of tobacco smoking and the unavailability of a separate kitchen on their child’s health. Promotions, in the form of subsidies, could be provided to disadvantaged sections to construct a separate room as a kitchen in the household. Further, maternal asthma as a risk factor for ARI indicates that a history of respiratory illness among family members can aid in the prevention and early diagnosis of the infection among children. The study also recommends making health centers more accessible to the people in terms of both proximity and costs.

Abbreviations: ARI- Acute Respiratory Infection; AOR- Adjusted Odds Ratio; CI- Confidence Interval; MICS- Multiple Indicator Cluster Surveys; NFHS- National Family Health Survey; SDG- Sustainable Development Goal; UT- Union Territory; WHO- World Health Organization; UN- United Nations; UNICEF- United Nations International Children’s Emergency Fund.

## Data Availability

The data for this research is available for the public in the DHS website at https://www.dhsprogram.com/.
